# Feasibility of biventricular 3D transthoracic echocardiography in the critically ill and comparison with conventional parameters

**DOI:** 10.1186/s13054-018-2133-7

**Published:** 2018-08-19

**Authors:** Sam Orde, Michel Slama, Nicola Stanley, Stephen Huang, Anthony Mclean

**Affiliations:** 1 Intensive Care Unit, Nepean Hospital, Sydney, 2750 Australia; 2 Intensive Care Unit, Nepean Hospital, Kingswood, Sydney, NSW 2749 Australia; 3 Medical ICU, Amiens University Hospital, Amiens, France; 4 ICU, St John of God Midland Hospital, Midland, WA 6056 Australia

**Keywords:** 3D, Echocardiography, Stroke volume, Critically ill, ICU

## Abstract

**Background:**

Transthoracic 3D cardiac analysis is enticing in its potential simplicity and wealth of data available. It has been suggested to be accurate vs magnetic resonance imaging in relatively stable patients, but feasibility and agreement with conventional echocardiographic assessment of stroke volume (SV) have not been thoroughly assessed in critically ill patients, who are traditionally harder to image. The objectives of this study were to compare 3D transthoracic volumetric analysis vs Doppler assessment of SV (which is suggested to be accurate in the critically ill) and Simpson’s biplane assessment in a cohort typical of the intensive care unit (ICU), where accurate assessment is important: mechanically ventilated patients with a significant ventilation/perfusion (V/Q) mismatch. We hypothesised that it would be feasible but might lack agreement.

**Methods:**

Patients were imaged within 24 hours of admission. Inclusion criteria were adult patients, V/Q mismatch present (defined as a ratio of arterial oxygen partial pressure to fractional inspired oxygen < 300), and mechanically ventilated with Doppler SV assessment possible. Biventricular echocardiographic volumetric analysis was performed using Siemens SC2000 along with standard Simpson’s biplane and Doppler SV assessment. 3D images were unacceptable if two segments or more were unable to be seen in two volumetric planes. 3D left ventricular (3DLV) and 3D right ventricular (3DRV) analyses were performed with the Tomtec Imaging and Siemens Acuson platforms, respectively.

**Results:**

Ninety-two patients were included (83 in sinus, 9 in atrial fibrillation). 3DLV and 3DRV analyses were feasible in 72% and 55% of patients, respectively; however, they underestimated SV compared with Doppler by 2.6 ml (± 10.4) and 4.1 ml (± 15.4), respectively. Limits of agreement for 2D, 3DLV and 3DRV volumetric analysis techniques were large.

**Conclusions:**

3DLV and 3DRV volumetric analyses appear feasible (obtainable) in the majority of mechanically ventilated ICU patients. Compared with the Doppler method, 3DLV and 3DRV volumetric analyses underestimate SV. The large limits of agreement between the methods also cast doubt on their comparability. Given the scenarios in which SV analysis is required (e.g., assessment of cardiac performance), our study cautions against the use of 3D SV clinically.

## Background

Assessing ventricular size and function is the foundation for the diagnosis of cardiac dysfunction in the critically ill. Stroke volume (SV), end-diastolic volume (EDV), end-systolic volume (ESV) and ejection fraction (EF) are important when considering management in many circumstances, such as heart failure, fluid administration and effect of treatment. SV and cardiac output estimation using Doppler echocardiography (echo) has been suggested to have sufficient precision to be able to estimate cardiac output in the critically ill [1, 2], and although it is far from perfect, it is a standard method of assessment in many intensive care units (ICUs). Echo technology is advancing, and techniques such as 3D echo are now available which can potentially hold some benefits over conventional echo methods and provide additional data that may be important (e.g., strain, twist and torsion). 3D echo has been suggested to be time-saving, reproducible and accurate vs magnetic resonance imaging (MRI) [1]. This has not been reliably assessed in the critically ill.

**Fig. 1:**
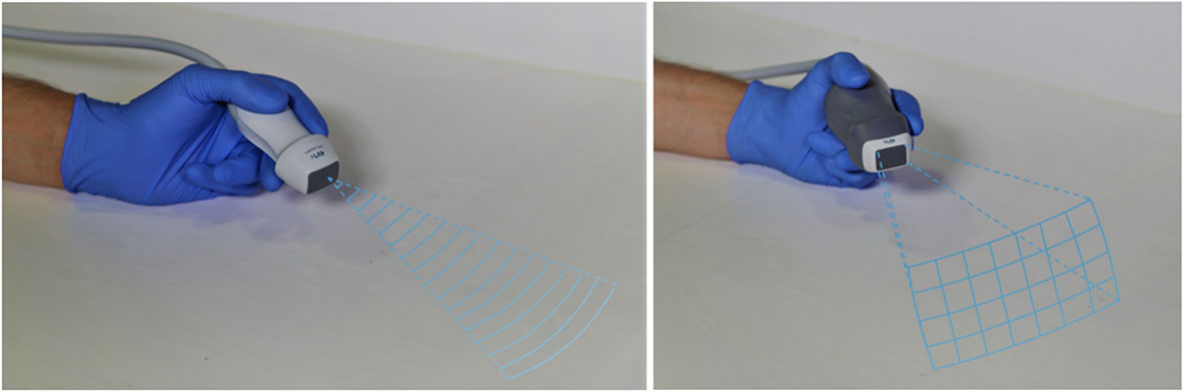
Two-dimensional (2D) [left] vs real-time three-dimensional (3D) [right] transthoracic echocardiography (echo). 2D echo provides single-plane assessment perpendicular to the piezoelectric crystal arrangement, whereas real-time 3D echo presents a pyramid or ‘volume’ of data

3D transthoracic echo has been a feature of most major ultrasound systems since 2008, being used for valvular analysis and volumetric and left ventricular (LV) mass estimation [2]. 3D left ventricular (3DLV) volumetric analysis with echocardiography is touted as more accurate than 2D echo volumetric estimation (using MRI as the gold standard) [3], and structures can be seen in the context of the whole myocardial volume rather than a single plane (*see* Fig. 1). EF, for example, can be hindered by foreshortening, malrotation or assumptions about ventricular shape, which may lead to inaccuracies. In addition, it is much more automated and may therefore provide rapid image analysis without additional human error or bias and has been shown to be repeatable in the cardiology setting [3].

3D volumetric measurements by echo were originally made by acquiring images over multiple heartbeats, obtaining the full-volume image through stitching together the data. More recently real-time 3D echo has been developed, which allows for the full volume to be recorded in one beat and prevents stitching artefacts, which can occur with respiratory movement or with arrhythmias such as atrial fibrillation. This can be particularly attractive in imaging the critically ill because breath holds can be challenging and arrhythmias are common. With real-time 3D transthoracic echo, there is reduced temporal and spatial resolution [4], and there is a need for specialised knowledge and equipment and importantly dependency on image quality.

We sought to assess if SV obtained by different methods, namely pulsed-wave Doppler, Simpson’s biplane and 3D echocardiography, is comparable and to assess if 3DLV and 3D right ventricular (3DRV) echo are feasible in an ICU population who were mechanically ventilated. We assumed that the SV of the LV would equal the SV of the right ventricle (RV). We chose a cohort of patients who would be considered typical ICU patients in whom ventricular volumetric analysis was important: mechanically ventilated critically ill patients with a significant ventilation/perfusion (V/Q) mismatch (defined by ratio of arterial oxygen partial pressure to fractional inspired oxygen [P/F], < 300).

## Methods

**Fig. 2:**
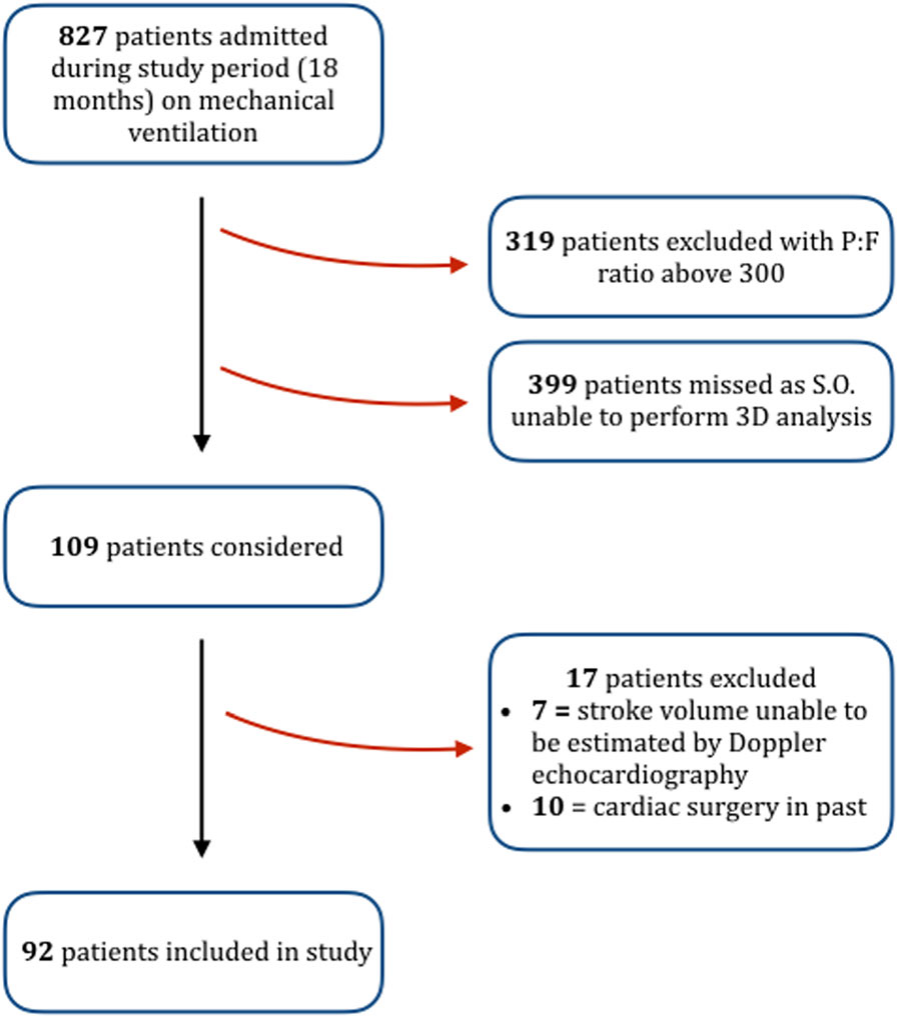
Study flowchart. *P:F* Ratio of arterial oxygen partial pressure to fractional inspired oxygen, S.O. Sam Orde (author) was primary person responsible for imaging

Adult patients admitted to the ICU of Nepean Hospital, Sydney, Australia, over an 18-month period were considered in this study. The project was approved by the Nepean Blue Mountains Local Health District (LNR/13/NEPEAN/154), and written consent was provided prospectively by the authorised representatives (next of kin) or retrospectively by the patient, given the non-invasive nature of the imaging. Patients were included if they were over the age of 18 years, were mechanically ventilated with a P/F ratio < 300 and were able to have SV assessed by Doppler echo. Patients were excluded if they had intracardiac shunts, previous cardiac surgery or congenital heart disease or were pregnant. We did not include consecutive patients, because SO was the sole investigator performing the 3D analysis and the majority of 2D studies (*see* Fig. 2 for study flowchart).

### Standard echocardiography

2D transthoracic echocardiography was performed by SO or research sonographers (all highly trained, fully qualified sonographers) using either a Vivid 7 machine (GE Medical systems, Chicago, IL, USA) with an M45 probe or a Siemens SC2000 with a 4V1c transducer (Siemens Healthineers, Erlangen, Germany). Accurate SV was ensured by estimating the left ventricular outflow tract (LVOT) diameter in a zoomed view of the LVOT and averaging LVOT velocity time integral measures with pulsed-wave Doppler (three cycles were averaged for patients in sinus rhythm and five for those in atrial fibrillation), with a closing click present and ensuring optimal Doppler angle and Doppler trace [5]. An LV centric apical four- and two-chamber view with minimised depth and optimal focal points was used to accurately estimate EF and volumes by Simpson’s biplane.

### 3D echocardiography

Real-time 3DLV and 3DRV assessment was performed using the 4Z1c full-volume 1.5–3.5-MHz matrix array transducer on the SC2000 echo machine by an experienced 3D operator (SO). The apical view was used, with the ventricle being analysed placed in the middle of the sector and the depth, sector size and angle adjusted to ensure maximal volumes per second (minimum acceptable 20 vol/s, range 20–45). Three cardiac cycles were recorded for sinus rhythm and five for atrial fibrillation. Images were analysed at stand-alone stations: LV images were transferred to a Tomtec system (TomTec Imaging, Unterschleissheim, Germany), and RV images were transferred to the SC2000 workstation using the RV analysis application; both systems use similar voxel analysis techniques and hence were felt to be comparable. Analysis was performed by clinicians with experience in 3D echo (SO and MS) using the automated analysis packages. SO completed all the offline 3D RV analyses, and MS completed all the offline 3D LV analyses. A 10% random population was assessed by both for inter- and intra-rater variability. Image quality was assessed in a manner similar to that in recent studies by reviewing the three planes that are provided. If two consecutive segments or more in any two views were not visualised, then the image was considered poor and unsuitable [6].

**Fig. 3:**
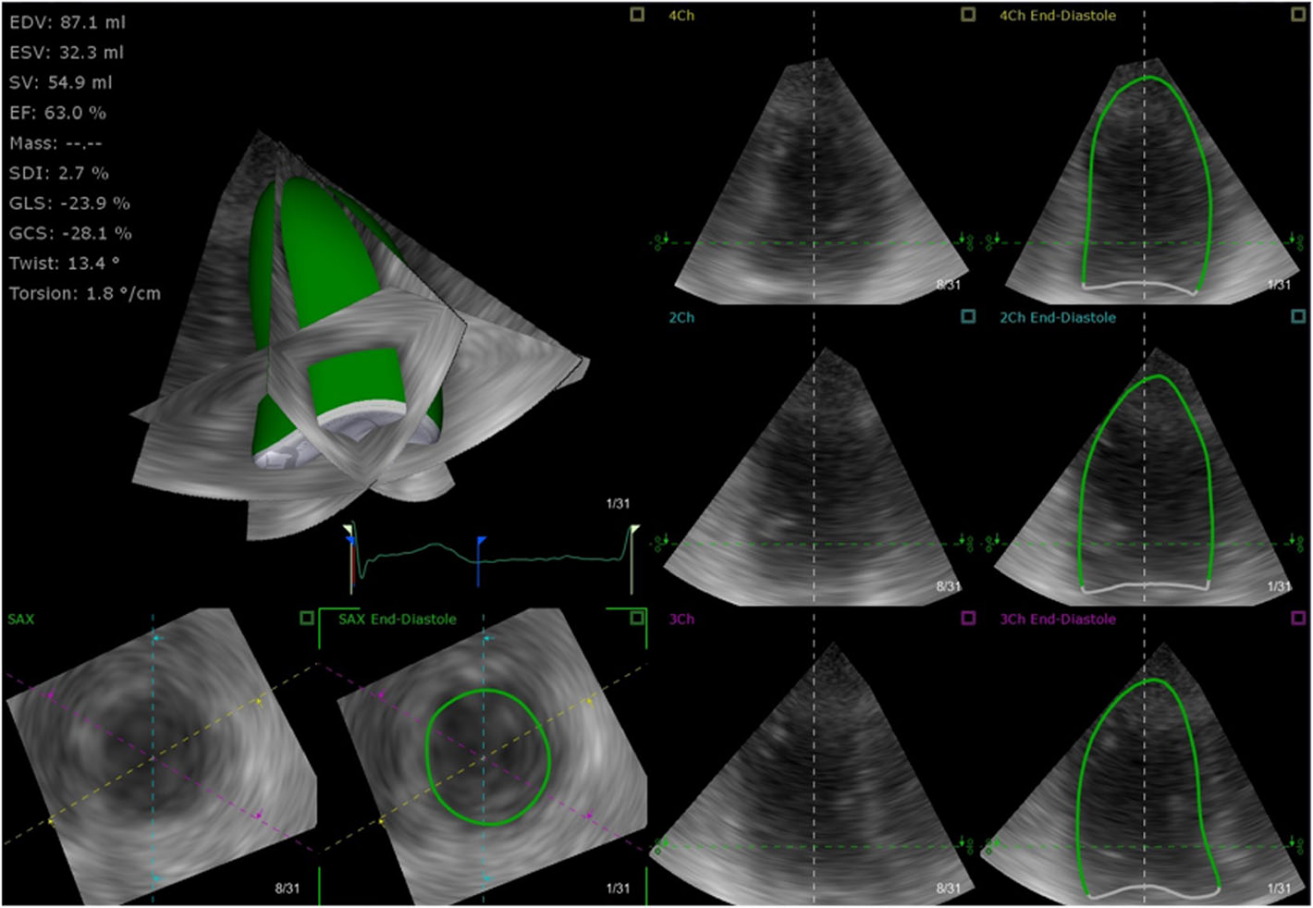
Real-time 3D left ventricular (LV) volume estimation with transthoracic echocardiography. The 3D LV volume is displayed (opaque green structure) with ‘cut planes’ provided in the short axis as well as four-, two- and three-chamber views. Semi-automated software estimates the endocardial border (*green line*) and its movement throughout the cardiac cycle. The user can alter this at end-systole and end-diastole if needed. *2Ch* two-chamber view, *3Ch* three-chamber view, *4Ch* four-chamber view, *EDV* End-diastolic volume, *EF* ejection fraction, *ESV* end-systolic volume, *GCS* global circumferential strain, *GLS* global longitudinal strain, *SAX* short-axis view, *SDI* systolic dyssynchrony index, *SV* stroke volume

**Fig. 4:**
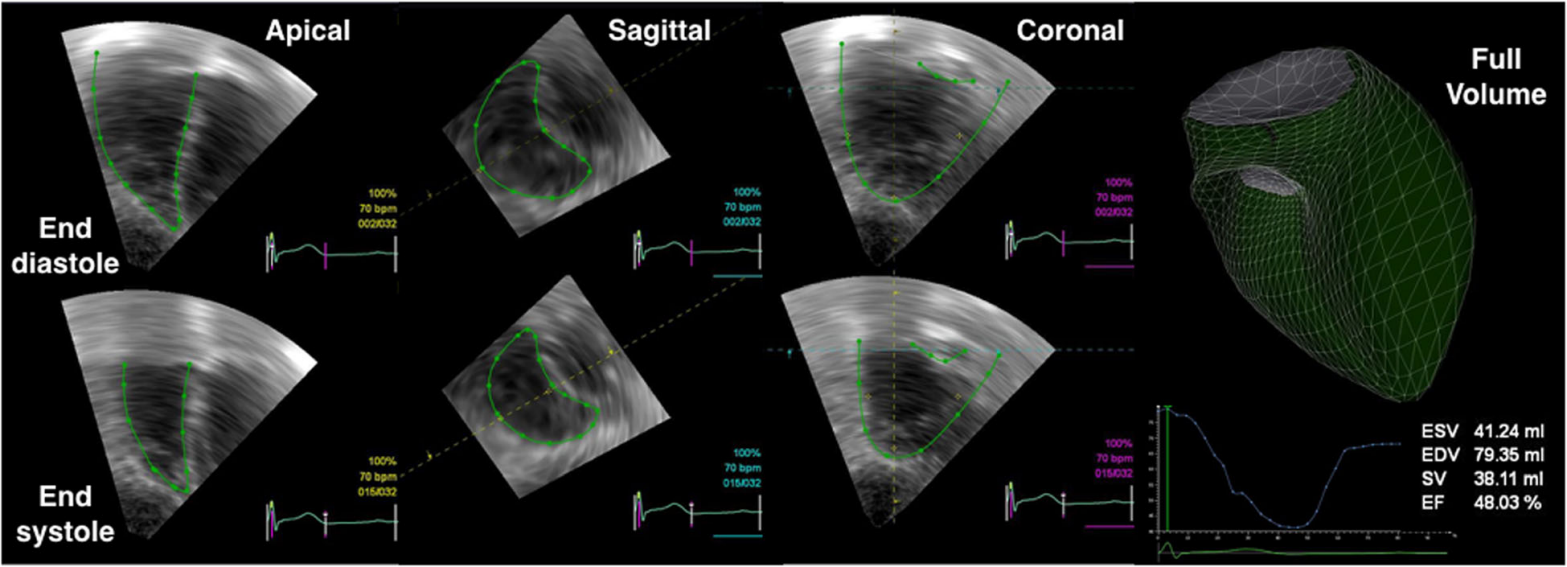
Real-time 3D right ventricular (RV) volume estimation with transthoracic echocardiography. *EDV* End-diastolic volume, *EF* Ejection fraction, *ESV* End-systolic volume, *SV* Stroke volume

The automated analysis packages were used to estimate volumes for both the LV and RV. LV volumes were estimated in end-diastole (ED) and end-systole (ES) by the software tracing the endocardium in three planes: apical four-, two- and three-chamber cut planes (*see* Fig. 3). The operator can perform changes to ensure accurate border identification in the images provided. The software then creates models of the LV cavity at ED and ES, from which the volumes (and other data) are estimated without making geometric assumptions. RV volumes use a similar principle of reviewing endocardial borders, but they need to be manually traced in the apical four-chamber, short-axis and coronal views in ED and ES, and volume change is then presented in an active 3D model (*see* Fig. 4). A method was considered feasible if it could be performed in the majority of patients included in the study.

### Statistics

Statistical analysis was performed with JMP Pro version 13 (SAS Institute Inc., Cary, NC, USA). Continuous variables are expressed as mean ± SD if normally distributed and as median with IQR if not normally distributed. Normality was assessed using the Shapiro-Wilk test. *P* values < 0.05 were considered statistically significant. Bias (mean of difference), precision (SD of difference) and limits of agreement (95% CI of the bias) statistics were performed using methods described by Bland and Altman [7]. To correct for magnitude-dependent variability, the bias was divided by mean SV and expressed as a percentage [8]. Thirty percent limits of agreement have been considered acceptable in previous meta-analysis data [9]. However, it should be noted that this ‘acceptable’ limit of agreement is based on the premise that both the reference method and the new method being investigated have percentage errors < 20%, whereas some recent evidence may suggest that both Doppler and 3D volumetric analysis may be greater [10, 11]. Feasibility for each analysis in this study was defined as the proportion of patients in whom the operator(s) could obtain optimal images for the respective analysis. Inter- and intra-rater variability was assessed by the absolute difference between 3D SV assessment methods vs Doppler and expressed as a percentage of their mean.

## Results

Ninety-nine patients were imaged, but seven patients were excluded because Doppler SV estimation could not be obtained reliably (7%). The characteristics of the 92 patients included in the study, along with ventilation data, are shown in Table 1. The majority of patients were in sinus rhythm and received mandatory mechanical ventilation. The patient group was critically unwell with a mean Acute Physiology and Chronic Health Evaluation III score of 84 (IQR 61–100) and were intubated for a median of 6 days. The most common reason for intubation was pneumonia (both community- and hospital-acquired). Median positive end-expiratory pressure levels were 8, with mean P/F ratios being in the moderate category by the Berlin definition of acute respiratory distress syndrome [12].

**Table 1:** Patient demographic and ventilation data from time of imaging (within 24 hours of admission to ICU)

Parameter			Value
Subjects, *n*			92
Age, years, median (IQR)			67 (57 to 73)
Sex, female, *n* (%)			42 (46%)
Rhythm, sinus rhythm, *n* (%)			83 (92%)
APACHE III score, mean (SD)			84 (61 to 100)
Ventilation time, days, median (IQR)			6 (3 to 9)
Diagnosis	Pneumonia		41 (45%)
	Aspiration		3 (3%)
	Cardiac		9 (10%)
	Abdominal sepsis (any source) with respiratory compromise		20 (22%)
	Exacerbation of COPD		11 (12%)
	Neutropaenic sepsis		3 (3%)
	Other		5 (5%)
Ventilation mode	Mandatory	Volume	63 (68%)
		Pressure	8 (9%)
	Pressure support		21 (23%)
P/F ratio, mean (SD)			175.5 (± 57)
PEEP, cmH_2_O, median (IQR)			8 (6.25 to 12)
Arterial oxygen saturation, %, median (IQR)			96 (91 to 97)

*Abbreviations: APACHE III* Acute Physiology and Chronic Health Evaluation III, *COPD* Chronic obstructive pulmonary disease, *P/F* Ratio of arterial oxygen partial pressure to fractional inspired oxygen, *PEEP* Positive end-expiratory pressure

The echo data are shown in Table 2 and include feasibility of each technique. Most patients were able to have Simpson’s biplane assessment with 2D imaging performed (85%), and the majority could have 3DLV assessment performed (72%); however, in only 55% of the patients included could 3DRV assessment be performed. 3DLV analysis took approximately 2–4 minutes, and 3DRV analysis 5–10 minutes, to perform per patient; these values are estimates only and were not formally timed.

**Table 2:** 2D and 3D echocardiographic data

Technique	Parameter	Feasibility, *n* (%)	Value, mean (SD) or median (IQR)
Doppler	Stroke volume	–	53.1 (17)
2D Simpson’s biplane	Stroke volume, ml	78 (85%)	53.0 (18)
	LV end-diastolic volume, ml		103.8 (86–137)
	LV end-systolic volume, ml		49.9 (33–73)
	Ejection fraction, %		52.6 (40–62)
3D Left Ventricle	Stroke volume, ml	66 (72%)	49.5 (35–59)
	LV end-diastolic volume, ml		97.7 (70–116)
	LV end-systolic volume, ml		50.7 (30–68)
	LV ejection fraction, %		51.5 (14.5)
3D RV stroke volume	RV stroke volume, ml	51 (55%)	43 (33–58)
	RV end-diastolic volume, ml		86 (72–125)
	RV end-systolic volume, ml		44 (33–67)
	RV ejection fraction, %		51 (42–56)

*Abbreviations: LV* Left ventricular, *RV* Right ventricular

### Stroke volume assessment

**Fig. 5:**
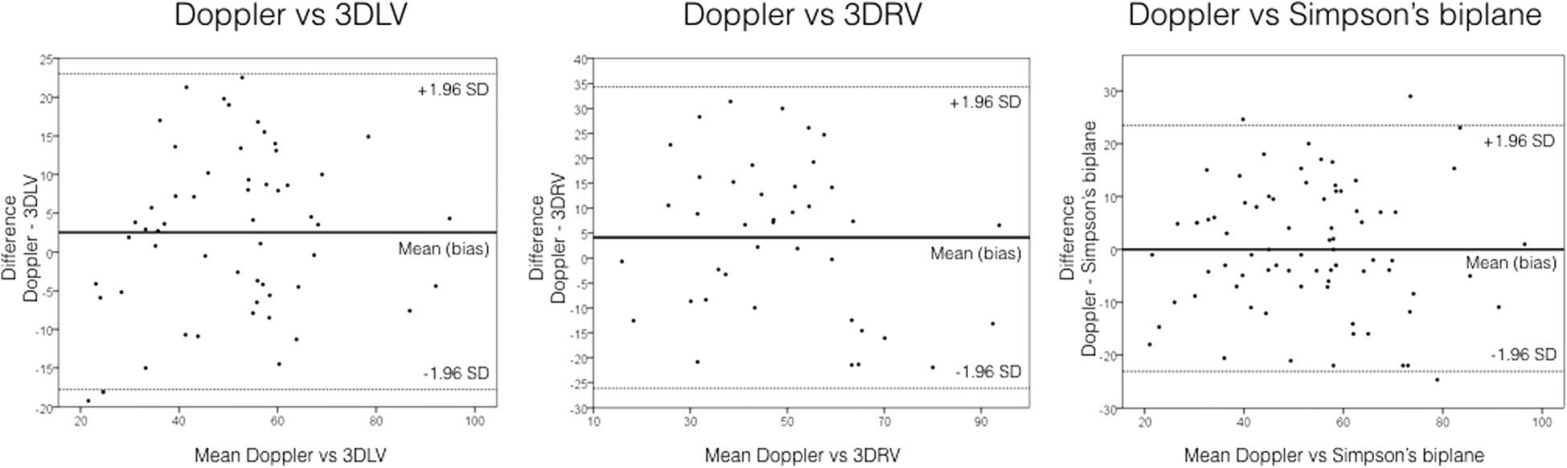
Bland-Altman plots comparing Doppler vs 3D left ventricular (LV) and 3D right ventricular (RV) stroke volume analysis as well as 2D ejection fraction stroke volume assessment by Simpson’s biplane method

Using Doppler as the reference method, 2D Simpson’s biplane, 3DLV and 3DRV analysis all underestimated SV (positive bias seen in Table 3), with 2D Simpson’s biplane assessment showing the smallest bias (0.2 ml) and 3DRV the greatest (4.1 ml). All three methods of SV assessment had wide ranges of limits of agreement (− 23 to 23 ml, − 18 to 23 ml, and − 26 to 34 ml for 2D Simpson’s biplane, 3DLV, and 3DRV, respectively, and corrected percentage errors of 50%, 51%, and 74%, respectively). Comparing 3DLV with 3DRV SV estimation, 3DRV analysis underestimated SV compared with 3DLV (bias 3 ml), again with wide limits of agreement (− 27 to 32 ml), and lacked agreement: The corrected percentage error was 40% (see Fig. 5).

**Table 3:** Bias, precision, limits of agreement and corrected percentage error between Doppler, 2D and 3D volumetric data

Techniques being compared	Value	Bias	Precision	Limits of agreement	Corrected Percentage error
Doppler vs 2D Simpson’s biplane	Stroke volume, ml	0.2	11.9	− 23.1 to 23.5	50.2%
Doppler vs 3D LV	Stroke volume, ml	2.6	10.4	− 17.8 to 23.0	51.3%
Doppler vs 3D RV	Stroke volume, ml	4.1	15.4	− 26.2 to 34.3	73.5%
3D LV vs 3D RV	Stroke volume, ml	2.7	14.9	− 26.5 to 31.9	67.6%
2D Simpson’s biplane vs 3D LV	Stroke volume, ml	2.8	11.5	− 19.7 to 25.4	40.1%
	LV end-diastolic volume, ml	5.7	19.7	− 33.0 to 44.3	37.0%
	LV end-systolic volume, ml	2.8	17.0	− 30.6 to 36.2	74.6%
	Ejection fraction, %	0.7	10.7	− 20.4 to 21.7	40.4%

*Abbreviations: LV* Left ventricular, *RV* Right ventricular

### Left ventricle assessment

Comparing 2D Simpson’s biplane and 3DLV assessments, 3DLV seemed to underestimate SV, LV end-diastolic and LV end-systolic volumes, and EF (bias 2.8 ml, 5.7 ml, 2.8 ml and 0.7%, respectively) with relatively wide ranges of limits of agreement (− 20 to 25 ml, − 33 to 44 ml, − 31 to 36 ml, and − 20 to 22%, respectively). The corrected percentage error was greatest for LV end-systolic volumes (75%) and was the smallest when comparing SV and EF (40%); however, it was still considered to lack clinical comparison.

### Repeatability

A random ten patients were selected for blinded variability analysis of the offline 3DLV and 3DRV SV assessments (i.e., analysis of the images). Inter-rater variability was reasonable for (1) 3DLV with mean absolute difference (±SD) of 3.6 ml (± 8.6) and expressed as percentage of the mean 8% (± 19) and (2) 3DRV with mean absolute difference (±SD) of − 2.1 (± 7.3) and expressed as percentage of the mean 10% (± 22). Intra-rater variability also showed reasonable repeatability for (1) 3DLV with mean absolute difference (±SD) of − 3.6 ml (± 8.8) and expressed as percentage of mean 7% (± 19) and (2) 3DRV with mean absolute difference (SD) of − 1.6 (10) and expressed as percentage of the mean difference of 4% (± 27).

## Discussion

We found real-time 3DLV and 3DRV transthoracic echo analysis of SV to be possible in a majority of critically ill patients, defined as patients on mechanical ventilation with significant V/Q mismatch; however, it did not have sufficient agreement with Doppler echo assessment to be considered clinically or statistically acceptable. In the intensive care clinical setting, cardiac volume analysis needs to be feasible, but low variability and high precision are key. We found that 3D transthoracic echo did not have sufficient agreement to be either statistically or clinically satisfactory for SV estimation in this study. 2D Simpson’s biplane analysis of SV was also assessed (because use of Doppler is considered to require higher levels of training [13]), and this also did not have sufficient precision to be considered acceptable vs Doppler analysis.

The ability to accurately measure SV in the critically ill plays an important role in analysis of cardiac function and haemodynamics, which are often abnormal in the ICU setting. Doppler echocardiography has been shown to be an accurate and precise method for estimating cardiac output and SV in the critically ill patient [10] and is the method of choice for many intensivists in cardiac assessment for fluid administration [14], evaluation of shock [15] and RV analysis [16]. 3D transthoracic echo transducers have become increasingly available and are described in the cardiology literature as having better accuracy and precision in measuring LV volumes than 2D transthoracic echo by methods such as Simpson’s biplane [17]. 3D echo may offer an advantage over conventional 2D echocardiography in a number of areas. In particular, the fact that the entire ventricle can be assessed rapidly, in a relatively automated fashion, means that errors such as angle dependence, as well as assumptions about the ventricle size or regional wall motion abnormalities, can be avoided. Imaging faults, such as foreshortening, which are reported to occur in approximately 50% of standard 2D imaging by sonographers, are avoided [18]. Compared with cardiac MRI, both 3D and 2D echo underestimate LV volumes. However, 3D under-represents values approximately 50% less than 2D and with approximately half the 95% CIs [17]. In addition, 3D transthoracic equipment is costly and requires significant training, and parameters such as diastolic function are not assessed.

There are several limitations to our study. It is a single-centre study performed by echocardiography enthusiasts. The 3D volumetric data were acquired on a single platform by a single operator, and data were analysed by the same operator, and therefore we cannot exclude bias. We attempted to limit bias by ignoring Doppler data prior to analysis of 3D volumes; however, a more structured blinding of data, or random assessment, would have meant greater scientific rigour. Data were analysed by a second experienced user and inter-rater variability was small and not statistically significant. The lack of consecutive assessment of patients indicates selection bias, but pragmatically, performing the study meant only one operator was regularly available for 3D imaging. The use of Doppler echo as the reference standard for SV estimation is controversial [19]. Basic evaluation of the errors of Doppler SV estimation vs thermodilution to guide sample size prior to starting the study would be ideal. In this regard, further studies using thermodilution, or ideally MRI, as the reference standard are warranted. In addition, we did not assess the trending ability of 3D transthoracic echo or the repeatability of the 3D data acquisition itself, and this may be a useful addition to future studies. Indeed, we postulate that the greatest source of variability in SV assessment using 3D transthoracic echo may be image acquisition itself. Finally, the reporting of limits of agreement may be considered controversial because larger limits of agreement may be considered statistically satisfactory due to previous evidence that both Doppler SV assessment and 3D volumetric analysis may have percentage errors > 20% vs a gold standard (thermodilution and MRI, respectively) [10, 11]. However, from a clinical perspective, tighter limits of agreement were felt to be more relevant.

Further studies are warranted in this area for analysis of precision (vs robust reference standards such as thermodilution of MRI), as well as in assessing the change in SV. In addition, comparison among groups of physicians with different levels of experience may be useful to confirm these results***.*** RV volumes in particular are not easily assessed by 2D echo, and given the complex shape of this ventricle and the extent of RV dysfunction in the critically ill, 3D RV analysis is enticing. 3D transoesophageal echo for volumetric analysis in the critically ill has been studied, particularly in the peri-operative setting, and has been suggested to be both feasible and as accurate as other forms of echocardiography [20]. Cardiac volumes and SV analysis play an important role in the care of the critically ill. Therefore, it is important to find a feasible, user-independent, repeatable, non-invasive, accurate technique in the critically ill.

## Conclusions

3DLV and 3DRV echo imaging in the critically ill is feasible and reproducible, but SV estimation by real-time 3D echo analysis did not have sufficient statistical or clinical agreement with Doppler evaluation of SV in this study.

## Abbreviations

2Ch: Two-chamber view
3Ch: Three-chamber view
3DLV: 3D left ventricular
3DRV: 3D right ventricular
4Ch: Four-chamber view
APACHE III: Acute Physiology and Chronic Health Evaluation III
COPD: Chronic obstructive pulmonary disease
ED: End-diastolic
EDV: End-diastolic volume
EF: Ejection fraction
ES: End-systolic
ESV: End-systolic volume
GCS: Global circumferential strain
GLS: Global longitudinal strain
ICU: Intensive care unit
LV: Left ventricle, left ventricular
LVOT: Left ventricular outflow tract
MRI: Magnetic resonance imaging
P/F, PaO_2_/FiO_2_: Ratio of arterial oxygen partial pressure to fractional inspired oxygen
P/F: Ratio of arterial oxygen partial pressure to fractional inspired oxygen
PEEP: Positive end-expiratory pressure
RV: Right ventricle, right ventricular
SAX: Short-axis view
SDI: Systolic dyssynchrony index
SV: Stroke volume
V/Q: Ventilation/perfusion
